# Treadmill training does not enhance skeletal muscle recovery following disuse atrophy in older male mice

**DOI:** 10.3389/fphys.2023.1263500

**Published:** 2023-10-24

**Authors:** Elena M. Yee, Carson T. Hauser, Jonathan J. Petrocelli, Naomi M. M. P. de Hart, Patrick J. Ferrara, Princess Bombyck, Zachary J. Fennel, Lisha van Onselen, Sohom Mookerjee, Katsuhiko Funai, J. David Symons, Micah J. Drummond

**Affiliations:** ^1^ Department of Physical Therapy and Athletic Training, University of Utah, Salt Lake City, UT, United States; ^2^ Department of Nutrition and Integrative Physiology, University of Utah, Salt Lake City, UT, United States; ^3^ Molecular Medicine Program, University of Utah, Salt Lake City, UT, United States

**Keywords:** aging, muscle regrowth, atrophy, inflammation, function, exercise

## Abstract

**Introduction:** A hallmark of aging is poor muscle recovery following disuse atrophy. Efficacious strategies to enhance muscle recovery following disuse atrophy in aging are non-existent. Prior exercise training could result in favorable muscle morphological and cellular adaptations that may promote muscle recovery in aging. Here, we characterized the impact of exercise training on skeletal muscle inflammatory and metabolic profiles and cellular remodeling and function, together with femoral artery reactivity prior to and following recovery from disuse atrophy in aged male mice. We hypothesized that 12 weeks of treadmill training in aged male mice would improve skeletal muscle cellular remodeling at baseline and during recovery from disuse atrophy, resulting in improved muscle regrowth.

**Methods:** Physical performance, *ex vivo* muscle and vascular function, tissue and organ mass, hindlimb muscle cellular remodeling (macrophage, satellite cell, capillary, myofiber size, and fibrosis), and proteolytic, inflammatory, and metabolic muscle transcripts were evaluated in aged exercise-trained and sedentary mice.

**Results:** We found that at baseline following exercise training (vs. sedentary mice), exercise capacity and physical function increased, fat mass decreased, and endothelial function improved. However, exercise training did not alter tibialis anterior or gastrocnemius muscle transcriptional profile, macrophage, satellite cell, capillarity or collagen content, or myofiber size and only tended to increase tibialis mass during recovery from disuse atrophy.

**Conclusion:** While exercise training in old male mice improved endothelial function, physical performance, and whole-body tissue composition as anticipated, 12 weeks of treadmill training had limited impact on skeletal muscle remodeling at baseline or in response to recovery following disuse atrophy.

## Introduction

Aging is characterized by systemic and localized tissue changes that increase the risk for multiple comorbidities. Sarcopenia is an age-related pathology that impacts skeletal muscle tissue and threatens the loss of independence, defined as the loss of muscle size, strength, and locomotor function with age (Coletta and Phillips, 2023). Like muscle aging, short-term muscle disuse (e.g., illness and injury following surgery) coupled with impaired muscle recovery can have profound effects on muscle mass and strength (Gallegly et al., 2004; Suetta et al., 2009; Hvid et al., 2010; Tanner et al., 2015; White et al., 2015; Baehr et al., 2016; Zhang et al., 2018; Oliveira et al., 2019; Reidy et al., 2019), thus further compounding age-related muscle dysfunction and physical disability. As such, there is a need to identify effective therapeutics that target defined dysregulated cellular events in aging muscle during regrowth following periods of disuse.

Effective muscle remodeling during recovery from disuse requires synchronized interaction among many interstitial cell types to facilitate muscle regrowth, such as satellite cells, macrophages, and endothelial cells. Macrophage cell subtypes play critical roles in the stimulation of satellite cell proliferation and differentiation as well as fibrogenesis and angiogenesis (Tidball et al., 2020). Indeed, muscle macrophages are positioned closely to muscle capillaries (Snijders et al., 2017; Snijders et al., 2019) and satellite cells (Walton et al., 2019), suggesting an important level of communication between the cell types. Evidence, including work by us, suggests that aged muscle has a defective or delayed macrophage inflammatory functional response under a variety of conditions such as muscle regrowth following disuse (White et al., 2015; Reidy et al., 2019; Fix et al., 2021). Moreover, the satellite cell pool (Brack et al., 2005; Wang et al., 2019) and function (Rhoads et al., 2013; Mahmassani et al., 2021) are decreased with age, suggesting an overall blunted myogenic capacity with aging. Aged muscle is also recognized to have diminished muscle capillarization (Degens et al., 1993; Moro et al., 2019) and endothelial cell function (Scioli et al., 2014; Socha and Segal, 2018). Therefore, dysfunctional macrophage, satellite cell, and endothelial cell/angiogenesis responses to cellular stressors (e.g., reloading) likely contribute to impaired muscle remodeling during recovery in aging.

Despite age-related muscle cellular dysfunction, human and rodent skeletal muscle retains the ability to undergo adaptation to exercise training including muscle fiber hypertrophy and increased muscle capillarity and satellite cell content (Moro et al., 2019; Dungan et al., 2022; Jones et al., 2023). In older adults, aerobic exercise training increased muscle macrophage content which corresponded with muscle size (Walton et al., 2019). Moreover, we have shown that treadmill exercise training in aged mice improves vascular function (Cho et al., 2021; Cho et al., 2022). Similarly, voluntary wheel running when administered after muscle disuse (hindlimb unloading) promotes physical function, satellite cell abundance, and muscle recovery following disuse atrophy in young mice (Hanson et al., 2010; Brooks et al., 2018), while treadmill exercise pre-conditioning in aged mice was capable of enhancing muscle regeneration (Joanisse et al., 2016). However, it is unknown whether prior exercise training in aged mice improve muscle size and cellular remodeling during recovery following disuse atrophy.

We used a progressive resistance treadmill training protocol previously demonstrated to affect cardiovascular, anthropometric, and skeletal muscle oxidative enzyme activity improvements in aged male mice (Cho et al., 2021). Therefore, we hypothesized that 12 weeks of treadmill exercise training in aged mice would improve muscle cellular content and remodeling (e.g., macrophages, satellite cells, capillary, collagen content, and fiber size), muscle transcriptional responses, and whole body and muscle function, resulting in enhanced muscle recovery following muscle disuse.

## Methods

### Animals

Seventy male C57BL/6 aged (19–20 months) mice were obtained from the National Institute on Aging mouse colony and used for the objectives described as follows. Animals were approximately 22–23 months of age when euthanized (roughly corresponding to a 65-year-old human (Tidball et al., 2020)). Animals were housed with *ad libitum* access to food and water and maintained on a 12:12-h light–dark cycle. All experimental procedures were conducted in accordance with the guidelines set by the University of Utah Institutional Animal Care and Use Committee.

### Exercise training

**FIGURE 1:**
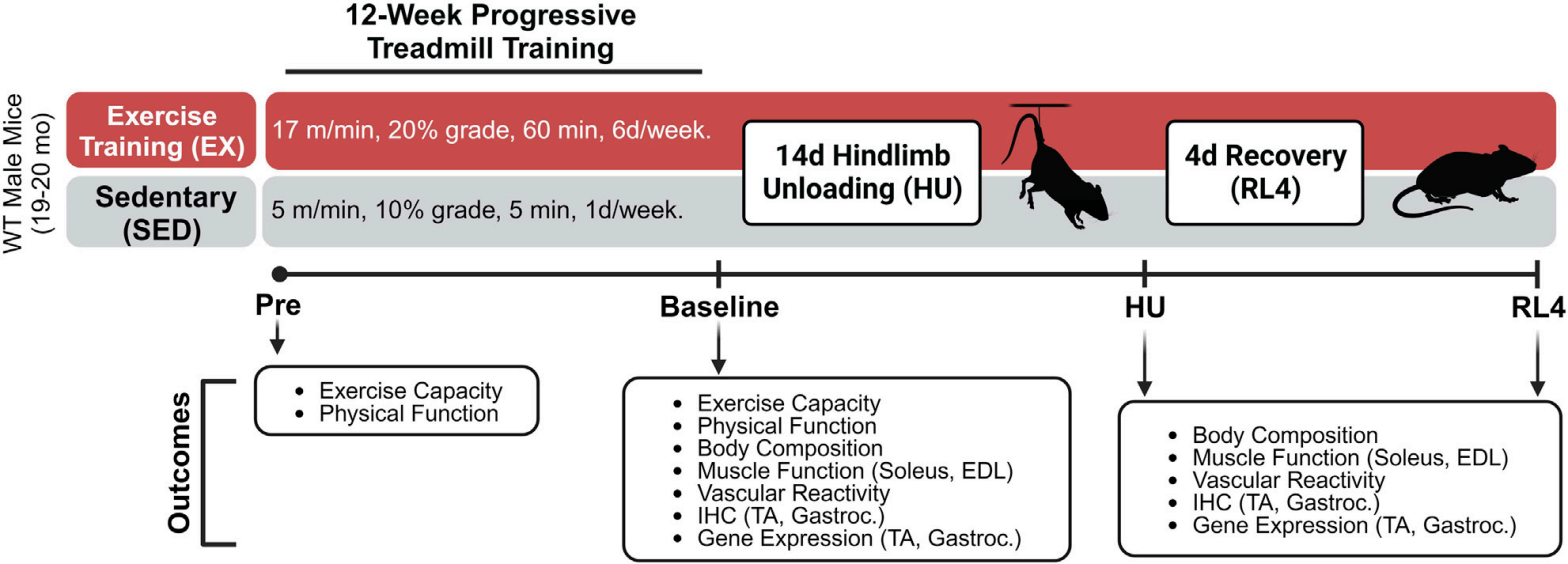
Treadmill exercise training experimental design. The schematic was created using Biorender.com under a paid subscription.

A schematic of the progressive treadmill exercise training protocol and experimental design can be found in Figure 1. Grip strength (Columbus Instruments, Columbus, OH), body weight, and physical function (Rotarod; Rotamex-5, Columbus Instruments, Columbus, OH) were assessed immediately before (pre) and after exercise training (baseline) in both exercise-trained (EX) and sedentary (SED) mice. Body composition (Bruker Minispec MQ20 nuclear magnetic resonance analyzer, Bruker, Rheinstetten, Germany) was assessed after exercise training and after 14 days of hindlimb unloading (HU) and at 4 days of recovery following HU (RL4). One week prior to treadmill familiarization and exercise training, pre-measurements were performed on mice. Following these measurements, the mice underwent a 4-day treadmill familiarization period in which they a) stood on the treadmill for 10 min at a 5% grade (days 1 and 2) and b) performed low-intensity exercise for 10 min at 6 m/min at a 5% grade (days 3 and 4), as we have done previously [33]. On the next day, an exercise capacity evaluation test was completed for each mouse. Mice started to exercise at 25% at 5 m/min for 1 min. After 1 minute, the speed was increased each minute by 1 m/min. This was repeated until each mouse reached its maximal exercise capacity. The total workload was calculated as (body weight (kg) x total running time (min) x final running speed (m/min) x treadmill grade (25%) (Cho et al., 2021). The maximal exercise capacity was defined by the inability of the mouse to maintain a smooth gait and/or failure to respond to the tapping of their rear using a test tube cleaning brush for encouragement.

Mice were then separated into groups that did not (SED) or did (EX) complete a 12-week (6 days/week) exercise training program, followed by assignment into either EX or SED baseline, hindlimb unloading (HU) and 4-day recovery (RL4) groups (Sed-baseline (n = 12), Sed-HU (n = 11), and Sed-RL4 (n = 12); Ex-baseline (n = 12), Ex-HU (n = 11), and Ex-RL4 (n = 12)). Those in the SED group completed a 12-week training protocol consisting of treadmill exposure for 1 day/week x 5 m/min x 10% grade for 5 min to maintain familiarization with the treadmill. When creating the exercise protocol, 70% of the mouse’s maximal speed was used to create a training regimen. Mice in the EX group initially ran at 70% of their maximal speed attained during the exercise capacity test (10 m/min) for 20 min at 5° incline. Exercise duration was increased every 4 days by 10–15 min until each mouse was running for 60 min x 10 m/min at 5° incline. After approximately 20 days, the speed was increased by 1 m/min and/or the incline was elevated by 5° at 4-day intervals until all mice were running for 60 min x 17 m/min at 20° incline. In some cases, when the treadmill incline was increased, the duration was decreased for several days in an effort to allow the mice to adjust to the new conditions (Supplementary Table S1). The mice were encouraged to complete each exercise session by tapping on their rear using a test tube cleaning brush. At the end of the 12-week training program, each mouse completed a post-program workload capacity evaluation test, and NMR, strength, and physical functions were re-tested. Mice were excluded during the 12-week exercise training regimen if they were no longer responding to the test tube brush tapping during the exercise training. In addition, mice were excluded if they became ill and could no longer participate in the training regimen. As a result, during the training period, seven mice in the exercise group and three in the sedentary group were excluded from the study.

### Hindlimb unloading and reloading

After 12 weeks of training, the baseline EX and SED controls were euthanized and endpoints were assessed as described further. The remaining EX and SED mice underwent 14 days of HU or 14 days of hindlimb unloading followed by 4 days of ambulatory recovery (RL4) based on their initial assignment. Mice in the HU and RL4 groups underwent 14 days of hindlimb suspension (two animals/cage) using a modified unloading method based on the traditional Morey–Holton design for studying disuse atrophy in rodents, as we have done previously (Reidy et al., 2019; Ferrara et al., 2022). Body weight and water and food intake were monitored every other day during HU to ensure that mice did not experience excessive weight loss due to malnutrition or dehydration. Following day 14 of HU, the animals in the RL4 group were removed from the suspension apparatus and housed in standard cages to undergo 4 days of reloading by ambulation. Four days of ambulatory reloading is an adequate time point of recovery in which we have previously observed muscle macrophage dysfunction in aged mice (Reidy et al., 2019; Fix et al., 2021; Ferrara et al., 2022). At baseline, day 14 of HU, or RL4, mice were starved (3 h), NMR was reassessed, and then mice were euthanized under isoflurane. Plantaris, soleus, gastrocnemius, extensor digitorum longus (EDL), TA, spleen, liver, heart, visceral, and epididymal fat were rapidly dissected, weighed, frozen in liquid nitrogen, or prepped in OCT and immersed in liquid nitrogen-cooled isopentane, and stored at −80°C for later analysis. *Ex vivo* muscle-specific force was determined in the soleus and EDL. The femoral artery was removed and used for vascular function analysis.

During hindlimb unloading, there were five SED and 11 EX mice lost due to illness ( significant decrease in body weight; died during HU). Therefore, n = 5 EX, and six SED mice were euthanized at the HU time point. Fewer mice were assigned to the HU group since we were primarily interested in the recovery from hindlimb unloading. Therefore, 11 sedentary and nine exercise-trained mice completed 4 days of reloading after HU.

### *Ex vivo* skeletal muscle force

Force production in soleus and EDL muscles were measured, as previously described (Petrocelli et al., 2021; Ferrara et al., 2022). Soleus muscles were sutured at each tendon and placed in a tissue bath (Aurora Scientific, Model 801C). Briefly, optimal length (L0) was reached through a series of pulse stimulations (0.2 ms pulse width, 20 V, 1 Hz). Rates of contraction and relaxation were measured as the average slope between 20% and 80% of the peak force of a pulse stimulation. Force–frequency relationship analyses were performed through stimulations ranging from 10 to 200 Hz (350 ms stimulation duration; 0.2 ms pulse width; 20 V) with 1 minute between each stimulation. A dual-mode lever force transducer (Aurora Scientific: 300 C-LR) with DMCv5.500 or DMAv5.321 software (Aurora Scientific) was used to measure force and analyze the data, respectively.

### Grip strength

To assess whole-body strength, mice underwent grip strength analysis on a rodent grip strength meter (Columbus Instruments, Columbus OH). Mice were acclimated to the procedure 1 week prior to exercise training. During this acclimation, mice were allowed to stand on the force transducer grid for a duration of 3 min. For the grip strength protocol, mice grasped the force transducer grid with their forelimbs and hindlimbs and were gently pulled by the tail across the grid. Five repetitions with a 5–10 s rest period were averaged to determine each animal’s grip strength. Grip strength was determined prior to exercise training and after exercise training.

### Rotarod

To assess balance and coordination, mice underwent rotarod analysis. A rotarod 3 cm in diameter, elevated to 16 cm above the instrument floor, divided into five equally spaced lanes was used. Individual trip plates were located on the instrument floor directly below each lane. Trip plates were used to stop the timer for each lane immediately upon contact with the mouse falling from the rod. The rotarod parameters were set at a start speed of 3 rpm and an acceleration rate of 25 rpm per 90 s. Mice were acclimated to the procedure 1 week prior to exercise training. During this acclimation, mice were allowed to stand on the rod for 3 min before initiating the rotation of the rod. For the protocol, mice were placed on the rod, and the machine began to track the speed and duration of the machine advancing at 25 rpm/min. Three repetitions with a 5–10 s rest period were averaged to determine the rotarod time and speed of each mouse. Rotarod speed and time were assessed prior to exercise training and after exercise training.

### Arterial function

Femoral arteries were cleaned of adherent tissue while bathed in an iced physiological saline solution (PSS) containing (mmol/L) 145.0 NaCl, 4.7 KCl, 2.0 CaCl_2_, 1.17 MgSO_4_, 5.0 glucose, 2.0 pyruvate, 0.02 EDTA, 3.0 MOPS buffer, 10 g/L of BSA at pH 7.4, and protease and phosphatase inhibitors. Each end of the vessel was cannulated using a glass micropipette tip with the aid of a dissecting microscope (SZX10; Olympus) (Cho et al., 2021; Cho et al., 2023). After the temperature of the bathing medium was increased over 30 min to 37°C, arteries were equilibrated for 1 h, followed by 10 mmHg increases in intraluminal pressure every 5 min to 60 mmHg. Four interventions separated by 30 min were observed in each vessel. First, a concentration–response curve to potassium chloride (KCl, 20–100 mmol/L) was obtained to measure non-receptor-mediated vasoconstriction. Second, to measure receptor-mediated vasoconstriction, a cumulative concentration–response curve to phenylephrine (PE, 10^−8^ M-10^−5^ mol/L) was completed. From these data, the dose of PE required to evoke 50% of maximal PE-evoked constriction was calculated. To evaluate endothelium-dependent vasodilation (third), an acetylcholine (ACh) dose–response curve (ACh, 10^−8^ M-10^−6^ mol/L) was completed in PE precontracted arteries, followed (fourth) by a sodium nitroprusside concentration–response curve (SNP, 10^−9^–10^−4^ mol/L) to assess endothelium-independent vasodilation. For ACh and SNP, percent vasodilation was calculated as (DT-Dp)/(Di-Dp) × 100, where DT is the recorded diameter at a given time point (i.e., diameter response to flow or SNP), Dp is the diameter recorded after the addition of the vasoactive agent (i.e., pre-constriction diameter), and Di is the diameter recorded immediately before the addition of the vasoactive agent (initial diameter). For KCl and PE, the percent vasoconstriction (% of baseline) was calculated as Dp/Di x 100.

*Citrate synthase (CS) activity* was assessed by using a colorimetric Abcam Citrate Synthase Assay Kit (ab239712). Flash-frozen gastrocnemius muscles were homogenized as outlined in the procedures from the kit. The samples were prepared and added to a 96-well plate where the reaction mix and background control mix were added to their respective well. Following plating, CS activity was assessed with a microplate reader (EPOCH, BioTek Instruments, Winooski, VT) at 412 nm by measuring the initial reaction rate of GSH followed by CS and CoA levels. For samples having a high CoA level, this amount was subtracted from total CS activity. From here, two time points were chosen based on the glutathione (GSH) standard curve. Sample activity was calculated using the equation given, followed by the normalization of CS to protein expression. After normalizing the data, CS activity was concluded.

### Immunohistochemistry

Frozen tibialis anterior and gastrocnemius muscle sections were used to determine the myofiber cross-sectional area (CSA) and myofiber-type, macrophage content, satellite cell content, capillary density, and fibrosis. For myofiber cross-sectional area and fiber-type identification, slides were incubated with WGA (AF350, 1:50, Invitrogen: W7024) to designate myofiber borders for assessment of myofiber-type-specific CSA, as we have done previously (Reidy et al., 2019). Myofiber CSA was measured using semiautomatic muscle analysis with segmentation of histology, a MATLAB application (SMASH) (Smith and Barton, 2014), alongside ImageJ software (Schindelin et al., 2012). For macrophage identification, anti-rat CD68 and anti-rabbit CD163 were utilized on sections. Anti-rat antibody (AF555, Thermo Fisher: B40933) and anti-rabbit (AF647, 1:250, Invitrogen: A21245) secondary antibodies were applied, and then the sections were incubated in 1:10,000 DAPI (Invitrogen: D3571) and 1:50 wheat germ agglutinin (WGA) (AF488, Invitrogen: W7024) for 10 min, followed by a mounting medium (Vector: H-1000). Satellite cell content was determined by treating the muscle section slides with primary antibodies for Pax7 and laminin. Nuclei were visualized with DAPI. Central nuclei were counted as nuclei not incorporated in the fiber border. To determine capillarization, slides were treated with primary antibodies for CD31 and laminin. For capillary identification, goat anti-rat Cy3 (1:250; Invitrogen: A10522) was utilized on sections. Nuclei were detected using DAPI. Collagen fibrosis content identification slides were first fixed in Bouin’s solution (LabChem, Zelienople, PA, United States, cat# LC117901), washed, and then incubated in Picrosirius Red solution (American MasterTech Scientific, Lodi, CA, United States, cat# STPSRPT) for 40 min. After washing and dehydrating in ethanol, the sections were dipped in xylene and mounted with CytoSeal XYL (ThermoScientific, Waltham, MA, United States, cat# 83124) to assess the collagen content (fibrosis). Nikon Elements Advanced Research software was used to analyze macrophage identification, satellite cell content, capillary density, and fibrosis. All slides were imaged using an Axio Scan.Z1 (Carl Zeiss Inc., Oberkochen, Germany) with a ×20 objective lens. Immunofluorescent stained slides were observed with the Axio Scan.Z1 attached to an X-Cite 120 LED Boost fluorescent laser.

### Gene expression

RNA was isolated from homogenizing the gastrocnemius (−35 mg) tissue by using the QIAzol lysis reagent (Qiagen 79,306), following the methods described previously (Petrocelli et al., 2021). To extract the RNA precipitate, chloroform, and isopropanol were used. After that, the precipitate was washed with 75% ethanol and then resuspended in nuclease-free water. The RNA concentrations were confirmed by using an EPOCH (Take3, BioTek, Winooski, VT, United States). Utilizing an iScript cDNA Synthesis Kit (Bio-Rad 17,088–91) and using a Bio-Rad T100 Thermal Cycler (settings: lid 105°C, volume 20 mL, 25°C 5 min, 46°C 20 min, 95°C 1 min, 4°C), 1 μg of RNA was reverse-transcribed. Real-time quantitative polymerase chain reaction was performed with diluted cDNA (in nuclease-free water) and SsoAdvanced Universal SYBR Green Supermix (Bio-Rad 17,252–70) on a CFX Connect real-time PCR detection system (Bio-Rad). All of the data were normalized to ribosomal protein L32. We chose gene expression readouts that were representative of muscle proteolysis and macrophage inflammatory and metabolic function as used by us and others (Reidy et al., 2019; Welc et al., 2020; Ferrara et al., 2022). The following primers were purchased from Bio-Rad: F-box protein 32 (Fbxo32, qMmuCED0045679), Forkhead box O3 (Foxo3a, qMmuCED0004522), hexokinase 2 (Hk2, qMmuCID0005994), interleukin 1 beta (IL-1β, qMmuCED0045755), interleukin 4 (IL-4, qMmuCID0006552), interleukin 6 (IL-6, qMmuCED0045760), interleukin 10 (IL-10, qMmuCED0044967), Kruppel-like factor 15 (Klf15, qMmuCID0008532), nuclear factor of kappa light polypeptide gene enhancer in B cells (NFκB1, qMmuCID0005357), prostaglandin–endoperoxide synthase 2 (COX2, qMmuCED0047314), tripartite motif-containing 63 (MuRF1, qMmuCID0014591), and tumor necrosis factor (TNF-α, qMmuCED0004141). Supplementary Table S2 provides designed primer sequences for CCL2, IGF-1, LDHA, PFKP, TGF-β1, and VEGFc that were purchased from the University of Utah Health Sciences Center Core.

### Statistical analysis

Statistical analysis was performed using Prism 7 software (GraphPad). Data were analyzed using a 2-way ANOVA (time and treatment). In case of the analysis of *ex vivo* contractile function and the arterial function independent variables were treatment and either frequency or concentration. The *post hoc* comparison test (Sidak’s) was performed when an interaction was detected. Baseline characteristics (workload capacity, rotarod, grip strength, and citrate synthase) were compared using a paired or unpaired *t*-test. All data represent mean ± SEM, and statistical significance was set at *p* < 0.05.

## Results

### Body composition, muscle, tissue, and organ weights

Lean and fat mass and fluid were examined using NMR. There was a main effect of time (*p* = 0.0238) and treatment (*p* = 0.0147) such that total lean mass was subtly higher for the exercise group. Exercise-trained mice had lower fat mass than sedentary mice after 12 weeks of training (interaction, *p* = 0.0102; *post hoc*, *p* < 0.0001) Table 1. Total body fluid was not different between groups. Body weight decreased in response to exercise training such that exercise-trained mice had a significant decrease in body weight compared to sedentary mice at baseline (interaction, *p* = 0.0022; *post hoc*, *p* = 0.0027).

Hindlimb muscle weights and select organs and tissues were weighed across time points for exercise and sedentary groups. The soleus weight changed over time (*p* < 0.0001) but was not different between the groups. When examining the TA weight, there was an interaction (*p* = 0.0090) such that the exercise group tended to have a lower weight at baseline and a higher weight at RL4 (∼*p* = 0.08). The gastrocnemius muscle changed over time (*p* < 0.0001) and treatment (*p* = 0.0271). There were no significant differences between the groups concerning EDL and plantaris muscle weight. Both the liver (*p* = 0.0150) and epididymal fat (eWAT) (*p* < 0.0001) were different over time. When combining the weights of the eWAT and inguinal fat (iWAT), a main effect of time (*p* = 0.0003) and treatment (*p* = 0.0476) was observed. There were no differences in spleen and heart weight, but the spleen weight for the exercise group was numerically lower at all time points when compared to sedentary mice at their respective time points.

### Exercise training performance and citrate synthase activity

**FIGURE 2:**
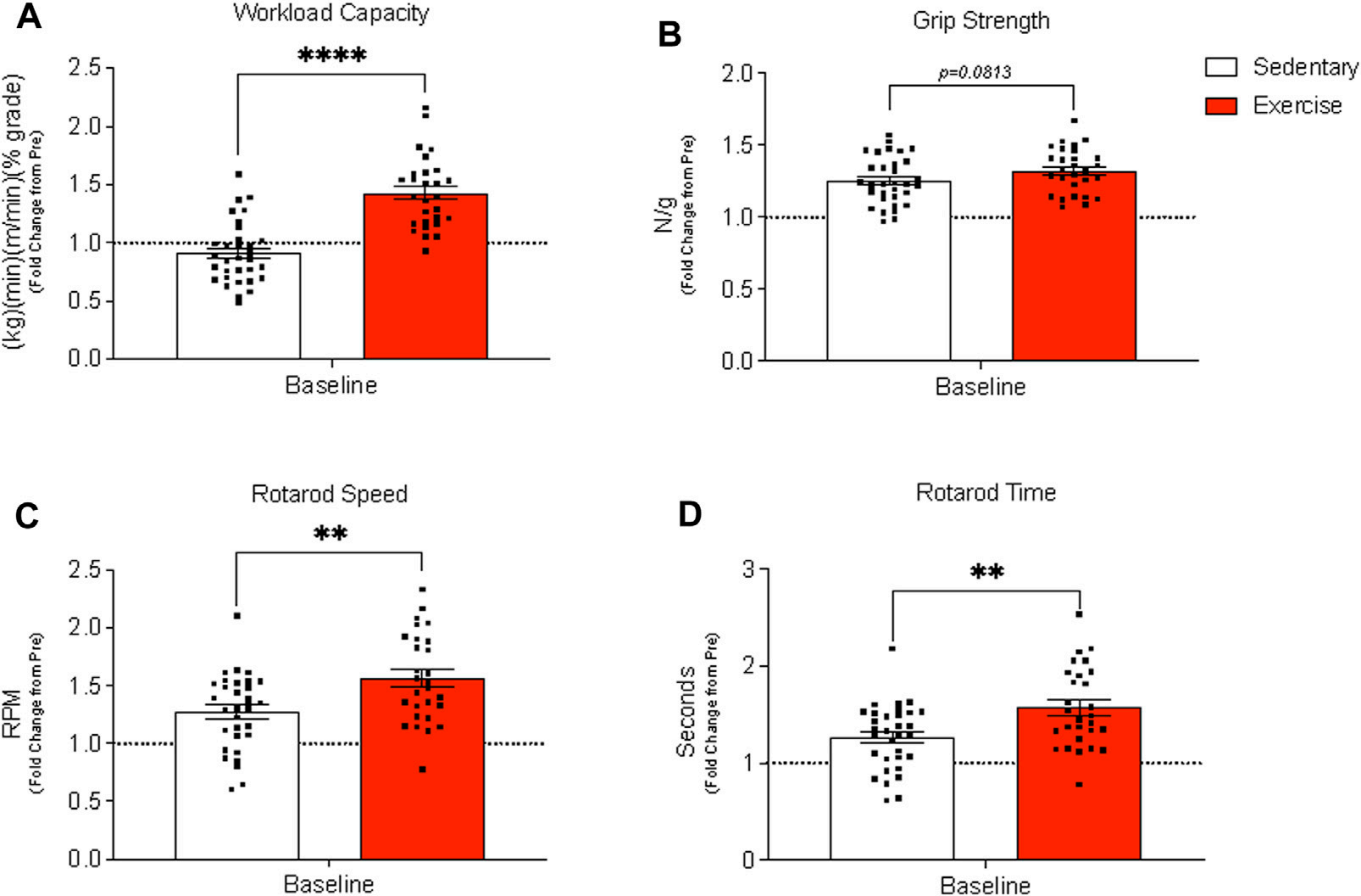
Assessment of whole-body physical function before (pre) and after (baseline) exercise training in exercise-trained and sedentary aged mice. **(A)** Workload capacity, **(B)** grip strength (normalized to body weight), and rotarod **(C)** speed **(C)** and time **(D)** were evaluated pre and post 12 weeks of treadmill training. Results are expressed as mean fold change from the pre-measurement with standard error of the mean. A paired *t*-test was used to compare sedentary and exercise-trained groups. ****p < 0.0001, **p ≤ 0.01. Panels **(A–D)** contain n = 28–32.

The exercise group, in comparison to the sedentary group, had a significantly higher workload capacity (Figure 2A) while grip strength (Figure 2B) tended to be higher (*p* = 0.0813) for the exercise group than for the sedentary group. Exercise training increased rotarod speed and time (Figures 2C, D), but citrate synthase activity in both TA and gastrocnemius muscles was unchanged at baseline between treatment groups (Supplementary Figures S1A, B).

### Arterial vasoreactivity

**FIGURE 3:**
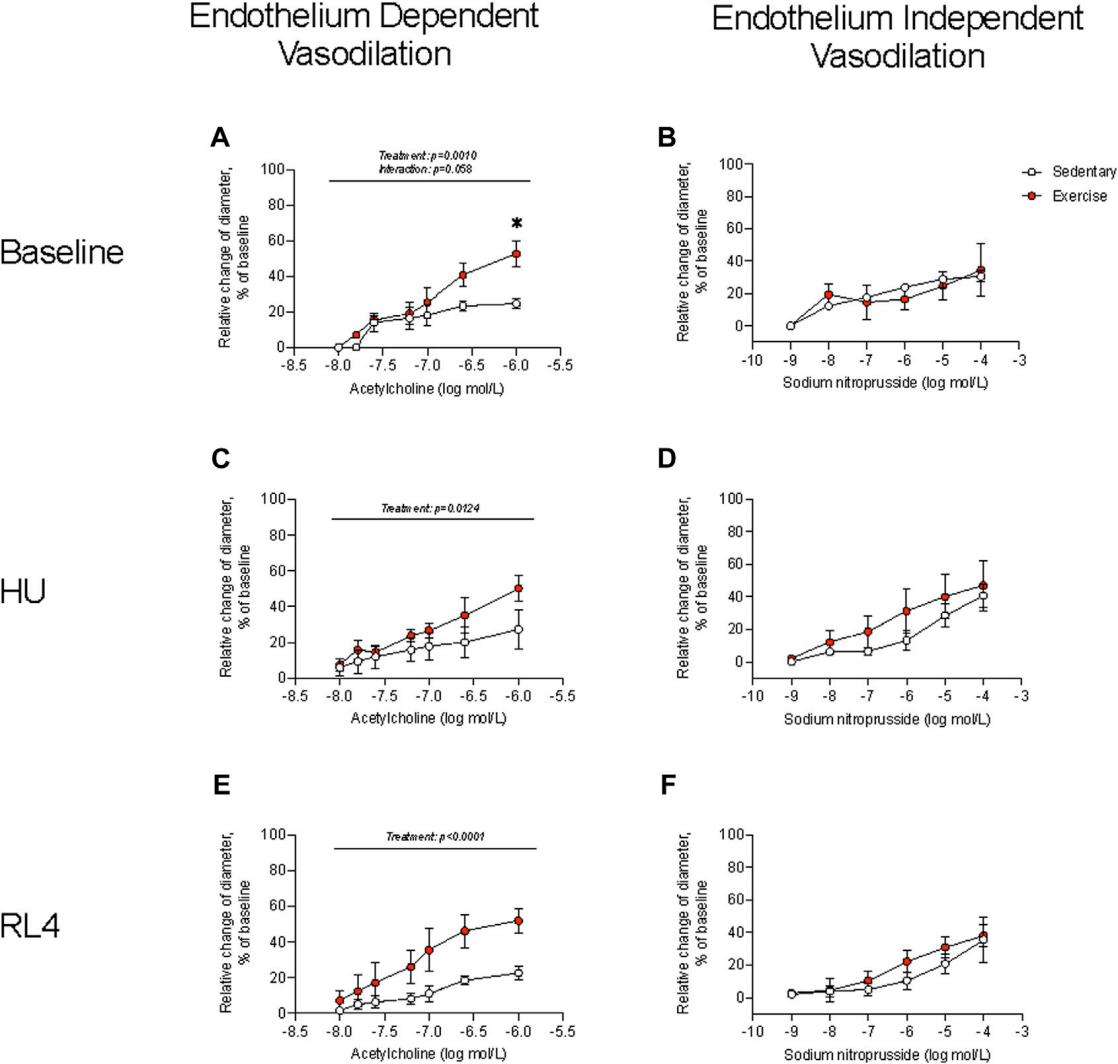
Endothelium-dependent vasodilation and endothelium-independent vasodilation were measured in sedentary and exercise-trained mice at baseline **(A, B)**, at hindlimb unloading (HU; **(C, D)**, and in response to 4 days of reloading (RL4; **(E, F)**. One femoral artery per mouse was evaluated. Baseline, n = 4; HU, n = 4; RL, n = 5. All data are presented as mean ± SEM. Results were compared using a two-way repeated measures ANOVA. A Sidak’s multiple comparisons test was used when an interaction was detected. *p < 0.05 sedentary vs. exercise at respective concentrations.

Femoral artery diameters at 0 and 60 mmHg were not different between sedentary and exercise-trained mice that completed the baseline, HU, and RL4 interventions. KCl depolarizes the vascular smooth muscle cell membrane to open voltage-gated Ca2+ channels (i.e., L-type Ca2+ channels). KCl-induced vasoconstriction was not different between sedentary and exercise-trained mice across time points (Supplementary Figure S2). PE binds to α1-adrenergic receptors that are coupled to Gq proteins. PE-mediated α1 activation stimulates inositol triphosphate-mediated release of Ca2+, which triggers Rho-kinase, inhibits myosin light-chain phosphatase, and evokes vascular smooth muscle constriction. While PE-induced vasoconstriction was not different between SED and exercise-trained mice that completed baseline and HU interventions, responses were blunted (*p* < 0.05) in arteries at RL4 between the two groups. ACh binds to M3 muscarinic receptors on the endothelium to increase intracellular Ca2+. Elevated intracellular Ca2+ then activates constitutive type III NOS, which enables conversion of the amino acid substrate L-arginine to the products L-citrulline and NO. NO then diffuses to the vascular smooth muscle where it activates guanylyl cyclase (GC). GC stimulation increases cGMP formation which inhibits Ca2+ entry into the vascular smooth muscle and precipitates vasodilation. Responses to these procedures indicated that endothelium-dependent vasodilation was greater (*p* < 0.05) in arteries from exercise-trained vs. sedentary mice across time points (Figures 3A, C, E). There was also a trend (*p* = 0.058) for there to be an interaction for endothelium-dependent vasodilation at baseline such that exercise-trained mice had higher vasodilation at the highest concentration (Figure 3A). Sodium nitroprusside (SNP) directly activates GC to evoke vasodilation that is independent of the endothelium. Responses to these procedures were not different between exercise-trained and sedentary mice at baseline, HU, or upon RL4 (Figures 3B, D, F), suggesting that exercise-mediated changes to arterial vasodilation occurred via endothelium-dependent pathways.

### *Ex vivo* muscle function testing

**FIGURE 4:**
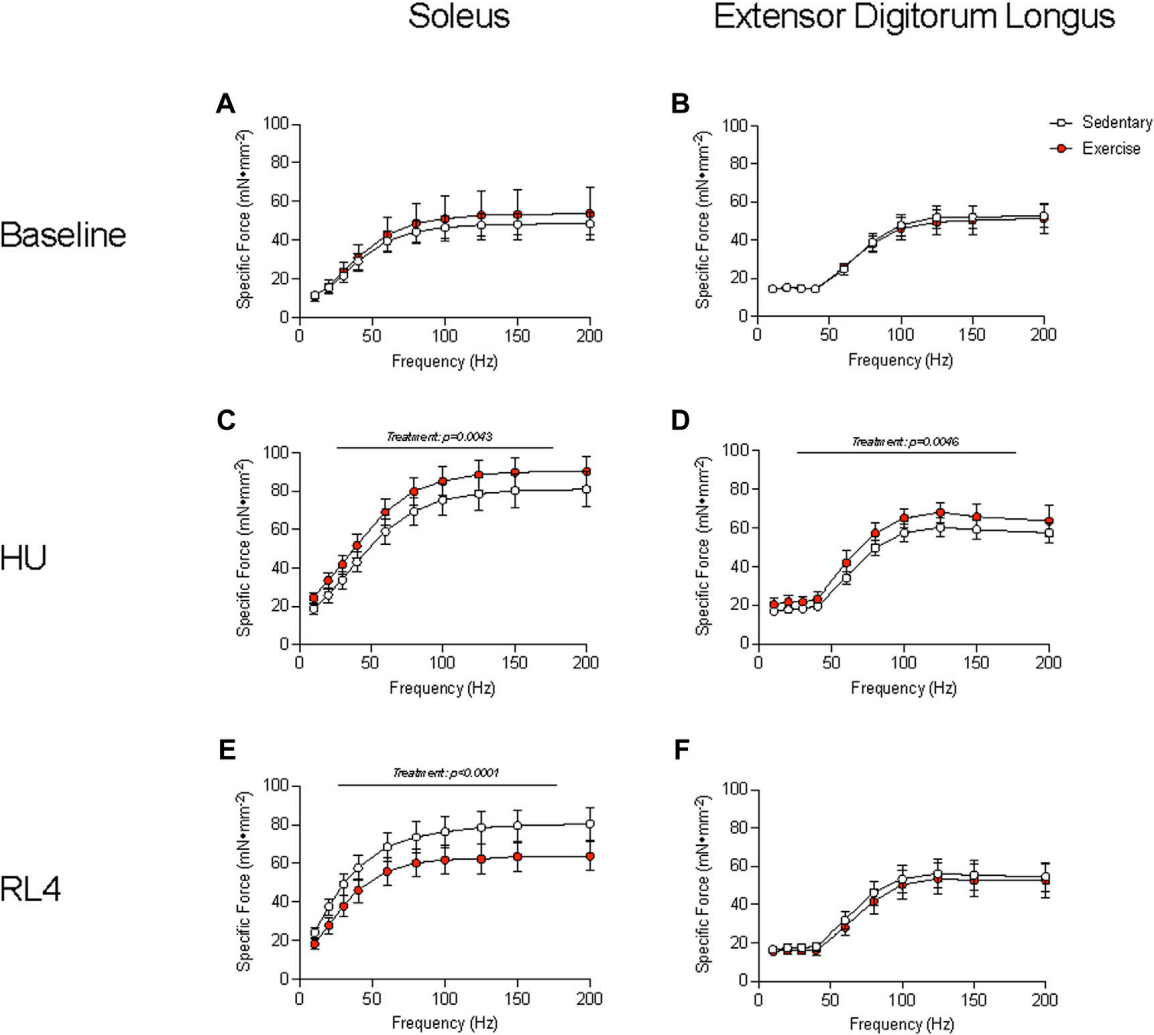
Muscle function of the soleus and the extensor digitorum longus (EDL) was examined by the specific force measured across a range (10–200 Hz) of stimulation frequencies in sedentary and exercise-trained groups. The force–frequency relationship was assessed at baseline **(A, B)**, HU **(C, D)**, and RL4 **(E, F)** in the soleus and the EDL. All data are presented as mean ± SEM. A two-way ANOVA was used. A Sidak’s multiple comparisons test was used when an interaction was detected. Panels A–F contain n = 3–10.

The soleus and EDL muscles were examined for *ex vivo* muscle function testing (force–frequency relationship) to determine how exercise could change contractile characteristics over a wide range of stimulation frequencies (10–200 Hz) and in different muscle groups. The force–frequency relationship of the soleus and EDL at baseline was not different between sedentary vs. exercise groups (Figures 4A, B). At the HU time point, the soleus and EDL (Figures 4C, D) exhibited a higher force–frequency relationship in the exercise-trained group (*p* = 0.0043). The soleus force–frequency relationship was lower for the exercise-trained group at the RL4 time point than for sedentary mice (Figure 4E), whereas that of the EDL was not different between the exercise and sedentary groups (Figure 4F).

### Tibialis anterior and gastrocnemius muscle myofiber CSA, macrophage immunofluorescence, satellite cells, capillary content, and muscle fibrosis/collagen accumulation

**FIGURE 5:**
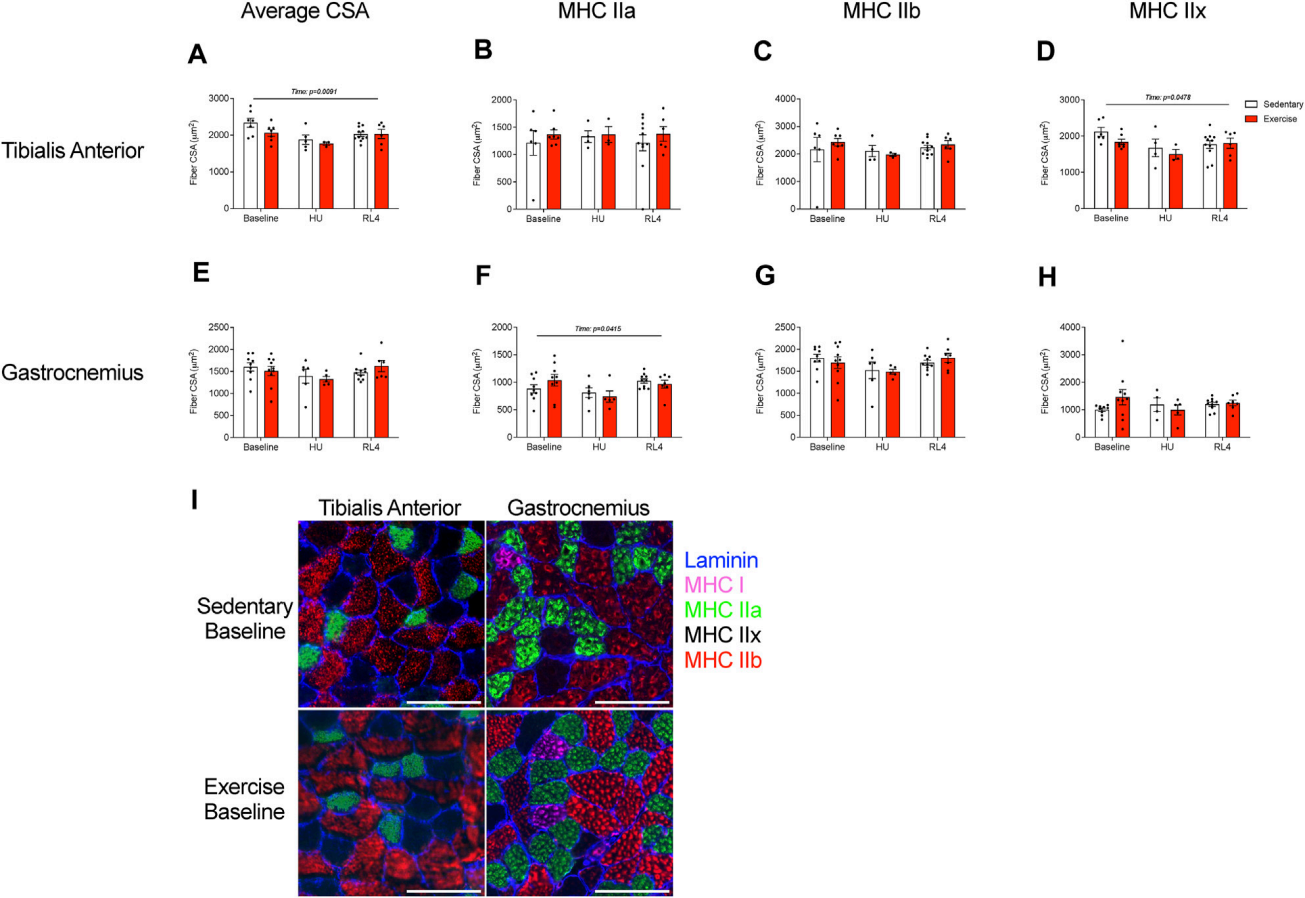
The fiber cross-sectional area (CSA) of the **(A–D)** tibialis anterior and **(E, F)** gastrocnemius was examined at baseline, HU, and RL4 time points for both sedentary and exercise groups. Both the tibialis anterior and gastrocnemius from all three time points (baseline, HU, and RL4) were sectioned and analyzed for **(A, E)** average CSA, **(B, F)** myosin heavy chain (MHC) type IIa fibers, **(C, G)** MHC type IIb fibers, and **(D, H)** MHC type IIx fibers. **(I)** Representative immunofluorescent cross-sectional images of the TA and gastrocnemius for baseline sedentary and exercise groups indicating fiber-type (blue = laminin, green = MHC IIa, red = MHC IIb, and negative = MHC IIx); scale bar represents 100 µm. All individual data points are presented as mean ± SEM. A two-way ANOVA was used. A Sidak’s multiple comparisons test was used when an interaction was detected. Panels **(A–H)** contain n = 3–10.

The fiber cross-sectional area (CSA) of the tibialis anterior and gastrocnemius muscles was evaluated in response to exercise training (baseline), HU, and recovery (RL4) from HU. Type 1 fibers were rare for TA and gastrocnemius muscle and, therefore, were not analyzed. For the TA, the average CSA (Figure 5A; *p* = 0.0091) was different over time, whereas MHC type IIa and IIb fiber CSA remained unchanged (Figures 5B, C). MHC type IIx fibers in the TA also exhibited differences over time (Figure 5D; *p* = 0.0478). For the gastrocnemius, the average CSA was not different (Figure 5E). However, MHC type IIa (Figure 5F; *p* = 0.0415) changed over time. There were no differences for gastrocnemius type IIb and IIx fiber CSA (Figures 5G, H). A representative image of fiber-type CSA in TA and gastrocnemius in sedentary and exercise groups at baseline can be found in Figure 5I.

**FIGURE 6:**
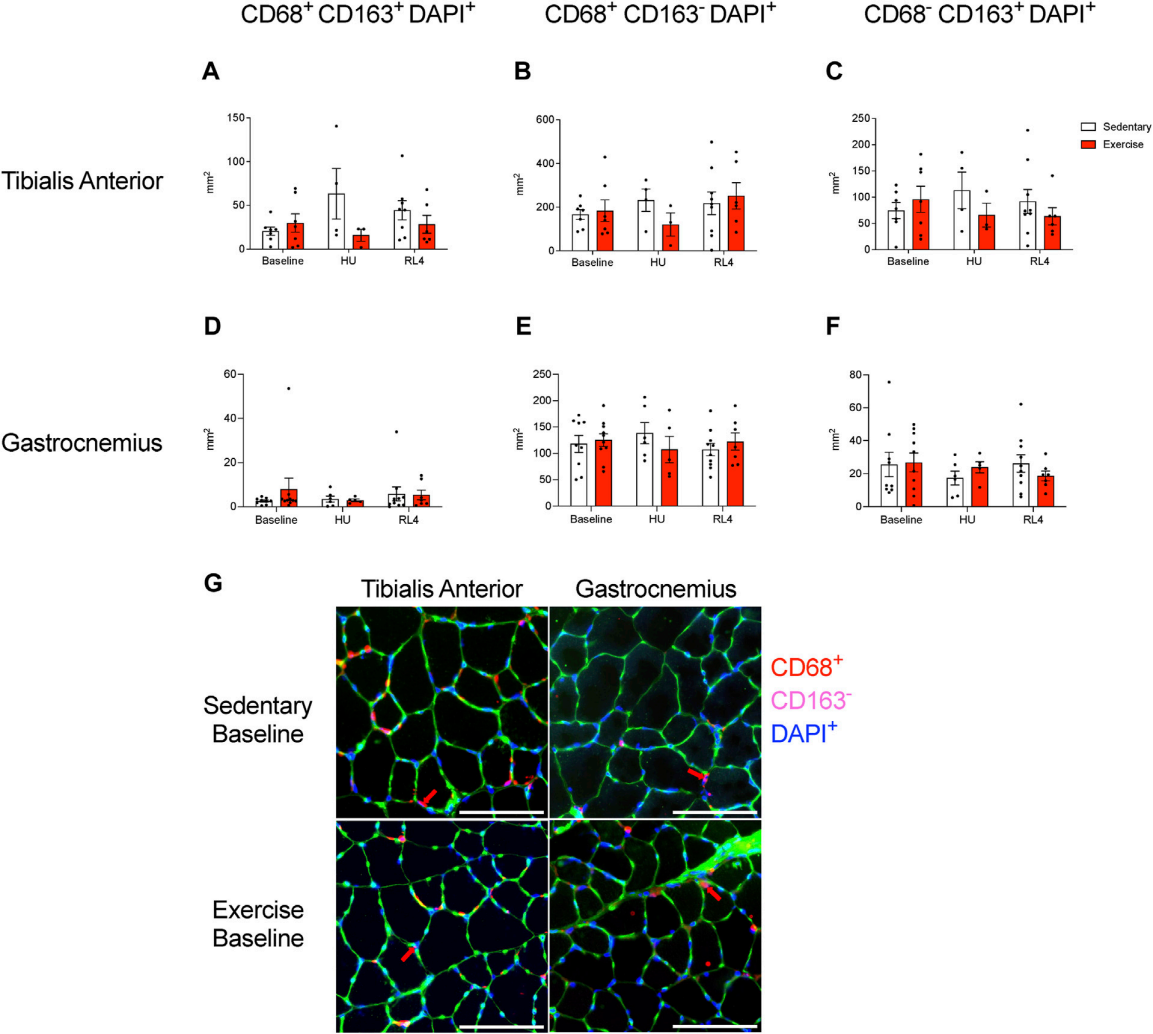
Macrophage immunofluorescence quantification was examined within the **(A–C)** tibialis anterior and the **(D–F)** gastrocnemius cross-sectional muscles for both sedentary (open bar) and exercise (red bar) groups at baseline, HU, and RL4 time points. The following inflammatory profiles were examined for macrophage abundance: CD68^+^ CD163^+^ DAPI^+^ macrophages, CD68^+^ CD163^−^ DAPI^+^ macrophages, and CD68^−^ CD163^+^ DAPI^+^ macrophages. **(G)** Representative immunofluorescent cross-sectional images of the TA and gastrocnemius muscles for both baseline sedentary and exercise groups (green = wheat germ agglutinin (WGA), blue = DAPI, red = CD68, and magenta = CD163 (low abundance); red arrows indicate cells that were positive for DAPI and CD68 but negative for CD163; scale bar represents 100 µm. All individual data points are presented as mean ± SEM. A two-way ANOVA was used. A Sidak’s multiple comparisons test was used when an interaction was detected. Panels A–F contain n = 3–10.

**FIGURE 7:**
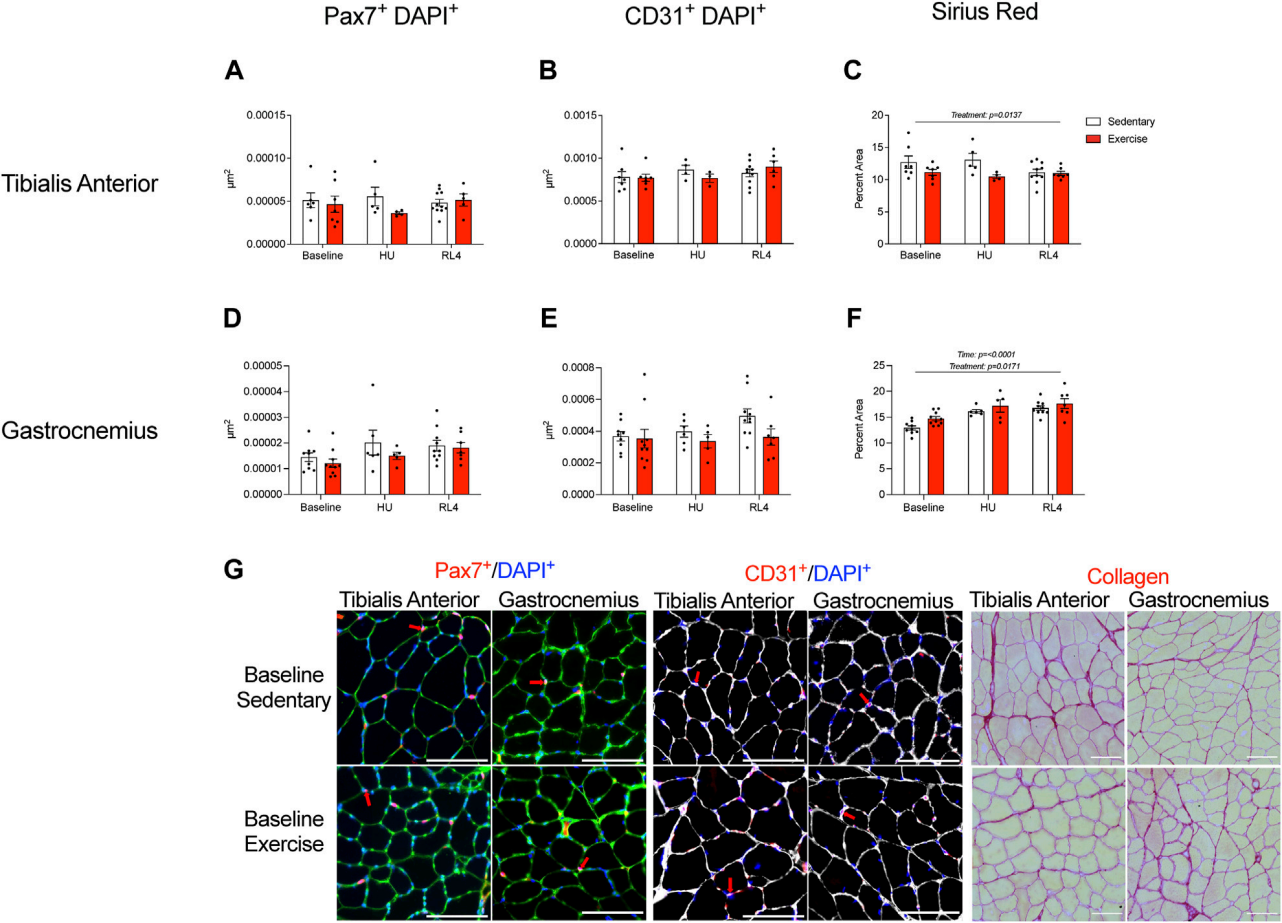
Satellite cells, capillary content, and muscle fibrosis/collagen were measured in the tibialis anterior **(A–C)** and gastrocnemius **(D–F)** cross-sections at baseline, HU, and RL4. Satellite cell immunofluorescence quantification was accessed by the overlay of Pax7^+^ and DAPI^+^ cells. Capillary content was determined from muscle cross-sections using the overlay of CD31^+^ and DAPI^+^ cells. The percentage of Picrosirius Red was used to determine muscle fibrosis in muscle cross-sections. **(G)** Representative immunofluorescent images of the quantified satellite cells, CD31^+^ cells, and collagen content (Picrosirius Red) found within the TA and gastrocnemius for baseline sedentary and exercise groups. The scale bar represents 100 µm. Colors represent the following: satellite cells: green = laminin, blue = DAPI, and red = Pax7; red arrows indicate positive DAPI and Pax7 cells. Capillary content: white = laminin, blue = DAPI, and red = CD31; red arrows denote positive DAPI and CD31 cells. Sirius Red: red = collagen and yellow = muscle. All individual data points are presented as mean ± SEM. A two-way ANOVA was used. A Sidak’s multiple comparisons test was used when an interaction was detected. Panels A–F contain n = 3–11.

Macrophage, satellite cell (Pax7^+^ and DAPI^+^), capillary (CD31^+^ and DAPI^+^), and collagen content (Sirius Red %) was examined in the cross-sections of the TA and gastrocnemius muscles in response to exercise training (baseline), HU, and recovery (RL4) from HU. CD68^+^, CD163^+^, DAPI, CD68^+^, CD163^-^, DAPI^+^, CD68^−^, CD163^+^, and DAPI^+^ macrophages in TA and gastrocnemius were not significantly different between exercise and sedentary mice across all time points (Figure 6). A representative image in TA and gastrocnemius in sedentary and exercise groups at baseline can be found in Figure 6G. For the TA, there were no significant differences in satellite cells and capillary content between the sedentary and exercise groups at all time points (Figures 7A, B); however, there was a main effect of treatment for Sirius Red % (Figure 7C; *p* = 0.0137). Similarly, there were no differences in satellite cell and capillary content for gastrocnemius muscle (Figures 7D, E). However, there was a main effect of time (Figure 7F; *p* < 0.0001) and treatment (*p* = 0.0171) in the gastrocnemius. A representative image of Pax7^+^ and CD31^+^ cells and Sirius Red in TA and gastrocnemius in sedentary and exercise groups at baseline can be found in Figure 7G.

### Muscle atrophy-related, inflammatory-related, and metabolic-related gene expression

**FIGURE 8:**
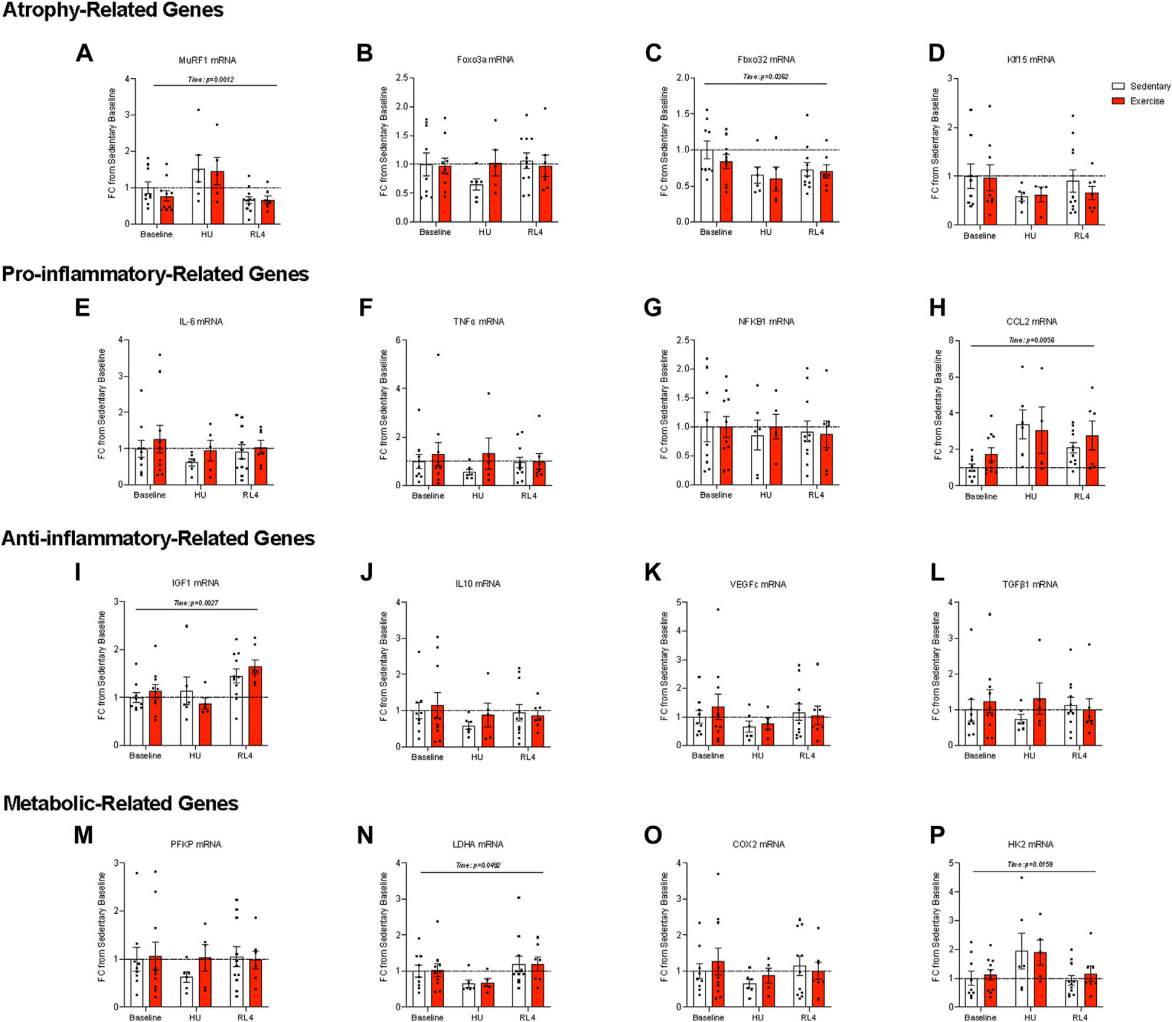
Gene expression of the gastrocnemius muscle at baseline, HU, and RL4. The following atrophy-related genes were accessed: **(A)** MuRF1, **(B)** Foxo3a, **(C)** Fbxo32, and **(D)** Klf15. Pro-inflammatory-related genes **(E)** IL-6, **(F)** TNF-α, **(G)** NF-κB1, and **(H)** CCL2 were examined. The following anti-inflammatory-related genes were evaluated: **(I)** IGF-1, **(J)** IL-10, **(K)** VEGFc, and **(L)** TGF-β1. Metabolism-related genes accessed are as follows: **(M)** PFKP, **(N)** LDHA, **(O)** COX2, and **(P)** HK2. All individual data points are presented as mean ± SEM. A two-way ANOVA was used. A Sidak’s multiple comparisons test was used when an interaction was detected. Panels A–F contain n = 4–11.

To determine if the muscle transcriptional landscape was altered due to exercise training, we measured the gene expression of select atrophy-related, inflammatory-related, and metabolic-related markers in the gastrocnemius muscle. MuRF1 (Figure 8A; *p* = 0.0012) changed over time, but Foxo3a remained unchanged (Figure 8B). Likewise, Fbxo32 (Figure 8C; *p* = 0.0362) was different across time points, while Klf15 mRNA was unaltered (Figure 8D). For the pro-inflammatory and anti-inflammatory-related genes, there were no differences in IL-6, TNF-α, and NFKB1 mRNA (Figures 8E–G), while CCL2 changed over time (Figure 8H; *p* = 0.0056). IGF1 mRNA changed over time (Figure 8I; *p* = 0.0027), but there were no changes in the mRNA abundance for IL10, VEGFc, or TGFB1 (Figures 8J–L). In regard to metabolism-related genes, PFKP mRNA (Figure 8M) was unaltered, yet LDHA (Figure 8N; *p* = 0.0492) changed across time. Likewise, COX2 mRNA (Figure 8O) was unchanged, while that of Hk2 (Figure 8P; *p* = 0.0159) changed across time.

## Discussion

This study aimed to determine if exercise training in aged mice would remodel skeletal muscle cellular and transcriptional responses that translate into improved muscle size and function at baseline and in response to recovery following disuse atrophy. As anticipated, exercise training in aged male mice (vs. sedentary age-matched controls) improved exercise capacity, whole-body function and tissue composition, and arterial vasodilation. Contrary to our hypothesis, skeletal muscle cellular remodeling, transcriptional changes, and muscle fiber size during recovery from hindlimb unloading were refractory to chronic exercise training. These data suggest that progressive treadmill training in aged male mice had limited impact on hindlimb muscle remodeling and regrowth following disuse atrophy despite notable training-induced effects on physical and vascular functions and body tissue composition.

Improved physical function and performance was an obvious outcome that supported aged male mice underwent favorable adaptation to 12 weeks of treadmill exercise training, as noted by increased rotarod performance and workload capacity and a trend for higher grip strength. Several studies (Seldeen et al., 2018; Kim et al., 2020) have shown exercise training-mediated increases in grip strength in young adult mice. Though we only observed a tendency for grip strength to increase following the exercise intervention, to the best of our knowledge, we are the first to report changes in rotarod performance after exercise training in aged male mice. Rotarod performance serves as an indicator of neuromuscular function, including balance, which overall declines with aging (Takeshita et al., 2017; Padilla et al., 2021). Exercise training in aged mice also resulted in important body composition changes, including decreased fat mass, which is a similar exercise training outcome observed in young adult mice (Kim et al., 2020). The change in fat mass tended to correspond with reduced epididymal and inguinal fat pad mass. Arterial vasodilatory function also improved with exercise training in aged mice and was endothelium-dependent as observed previously (Cho et al., 2021) but, collectively, arterial function was not acutely impacted by hindlimb unloading or recovery from disuse atrophy. Together, we show that moderate aerobic exercise not only reduces fat mass and increases vascular function but is also capable of improving balance and coordination in aged male mice.

Although treadmill exercise training in aged male mice resulted in whole-body tissue adaptations and improvements in physical and vascular function after exercise training, this did not translate into expansion of important muscle cellular pools (macrophages and satellite cells), improvements in soleus and EDL contractile function, remodeling events (capillarity and fiber size), or acute transcriptional responses critical for healthy aging and effective muscle regrowth. In agreement, Kayani and colleagues showed that aged male mice had limited muscle EDL contractile adaptation following 10 weeks of less intense treadmill training (15 m/min, 15 min, 3 days per week) (Kayani et al., 2008). Though treadmill training in aged male mice did not result in cellular remodeling, we cannot rule out that cellular function may have undergone favorable adaptation independently of morphological changes. For example, Brett et al., 2020 showed that as little as 3 weeks of voluntary wheel running in aged male mice resulted in improved muscle satellite cell activation. Perhaps if local cellular function was examined (e.g., muscle microvascular perfusion), the results may have been consistent with what we detected at the femoral artery and at the whole-body level.

Nonetheless, we were surprised that exercise training did not evoke more robust local muscle remodeling events in aged mice as reported by others. For example, Joanisse et al., 2016 noted that 8 weeks of treadmill exercise training in aged male mice increased tibialis anterior muscle capillary density and satellite cell abundance compared to sedentary age-matched controls. We believe the contrasting findings of our work compared to those of the Joanisse study may be due to differences in the exercise protocol administered to aged male mice. The protocol in our study included a higher volume of exercise (12 (current study) vs. 8 weeks; 6 (current study) vs. three times a week) and incorporated an incline, which was overall not the case for Joanisse et al., 2016. Joanisee and colleagues also used cardiotoxin injury to demonstrate improved muscle regeneration in aged mice following exercise training, while we employed disuse atrophy followed by ambulatory recovery, which induces much less muscle damage (Tidball et al., 1999; Kanazawa et al., 2017). We also cannot rule out that the volume of exercise (60 min/day, 6 days/week for 12 weeks) with minimal rest days (1 day/week) employed in the current study may have been too high in aged male mice to elicit local muscle changes, given that gastrocnemius muscle fibrosis was higher and soleus muscle contractile properties were lower during recovery. Indeed, prior work showed that treadmill overtraining in mice (75 min/d, 75% exercise capacity, 5 days/wk, for 8 weeks) blunted hypertrophic cell signaling in young adult C57Bl6 mice (da Rocha et al., 2016). Moreover, muscle citrate synthase levels in TA and gastrocnemius after exercise training was unchanged (vs. sedentary) in our study, suggesting that mitochondrial enzymes associated with local aerobic adaptations did not occur as we originally anticipated. More in-depth mitochondrial phenotyping may be necessary to fully validate adaptations to aerobic exercise and should be considered in future aging and atrophy studies (Calvani et al., 2013). Moreover, exercise volume should be considered in designed optimal exercise training studies in aged male mice.

Another important aspect of our study that may explain why treadmill exercise was ineffective to alter cellular remodeling and muscle regrowth in aged mice was that the chosen mode of exercise training lacked a resistance exercise component, which may be necessary to stimulate hypertrophy and alter muscle intracellular compartments critical for regrowth in aging. Indeed, in our study, hindlimb muscle mass (TA, gastrocnemius) tended to decrease in exercise-trained aged mice (vs. sedentary controls). This is in contrast to a recent study by Dungan et al., where the authors showed that 8 weeks of progressive voluntary wheel running with magnet-induced drag in aged female mice resulted in increased soleus muscle mass and soleus and plantaris capillary density, satellite cell content, and fiber cross-sectional area (Dungan et al., 2022). Though we cannot rule out that exercise training-mediated sex differences may partly explain the discordant findings between our study *versus* those of Dungan and workers (Triolo et al., 2022), we suggest that it might be necessary to incorporate a pre-conditioning exercise training regimen built around resistance-type exercise to enhance recovery from disuse atrophy in aging (Olesen et al., 2021; Dungan et al., 2022). We also warrant future studies to consider incorporating exercise during ambulatory recovery following disuse atrophy in aged mice since prior studies support that wheel running exercise following hindlimb unloading in sedentary young adult mice enhanced muscle recovery (Hanson et al., 2010; Brooks et al., 2018).

Finally, we would like to point out that the TA muscle mass was higher during early recovery from hindlimb unloading in the exercise-trained mice (vs. sedentary), but this did not translate to changes in the fiber cross-sectional area or lower content of non-contractile tissue (fibrosis). This suggests that the increased weight of the muscles may be due to water retention (edema; numerically higher at the whole-body level). However, we did not have the resolution in our measurement to statistically analyze this case (water content); thus, it would likely require the measurement of wet vs. dry weight of the muscle. Together, these data suggest that exercise training increased the muscle size of the TA muscle during recovery, but this was not related to fiber size or collagen content.

In conclusion, we showed evidence that exercise training in aged mice improved whole-body tissue composition, vascular function, and physical performance. However, 12 weeks of treadmill running was not effective to robustly alter cellular remodeling of the hindlimb muscles and enhance muscle size recovery in aged male mice. Future studies are recommended to evaluate the role of resistance exercise pre-conditioning on muscle regrowth in aging while also considering the impact of exercise volume and sexual dimorphism.

## Data Availability

The data that supports the findings of this study is available in the Supplementary Material of this article. Raw data will be made available upon request.

## Supplementary material

SUPPLEMENTARY FIGURE S1 Receptor-independent vasoconstriction and receptor-dependent vasoconstriction were measured in exercise-trained and sedentary mice at baseline **(A, B)**, and upon HU **(C, D)** or reloading (RL4; **E, F**). One femoral artery per mouse was evaluated. Baseline, n = 4; HU, n = 4; RL4, n = 5. Baseline, *n* = 4; HU, *n* = 4; RL, *n* = 5. All data are presented as mean ± SEM. Results were compared using a two-way repeated measures ANOVA. A Sidak’s multiple comparisons test was used when an interaction was detected. **p* < 0.05 sedentary vs. exercise at respective concentrations.

SUPPLEMENTARY FIGURE S2 Citrate synthase activity was measured in the TA **(A)** and gastrocnemius **(B)** at the baseline timepoint for both the sedentary and exercise groups. An unpaired t-test was used to compare sedentary and exercise trained groups. **p* < 0.05. Panel **(A)** and **(B)** contains *n* = 9.

## References

[B1] Baehr L. M. West D. W. Marcotte G. Marshall A. G. De Sousa L. G. Baar K. (2016). Age-related deficits in skeletal muscle recovery following disuse are associated with neuromuscular junction instability and ER stress, not impaired protein synthesis. Aging (Albany NY) 8, 127–146. 10.18632/aging.100879 26826670 PMC4761718

[B2] Brack A. S. Bildsoe H. Hughes S. M. (2005). Evidence that satellite cell decrement contributes to preferential decline in nuclear number from large fibres during murine age-related muscle atrophy. J. Cell Sci. 118, 4813–4821. 10.1242/jcs.02602 16219688

[B3] Brett J. O. Arjona M. Ikeda M. Quarta M. De Morree A. Egner I. M. (2020). Exercise rejuvenates quiescent skeletal muscle stem cells in old mice through restoration of Cyclin D1. Nat. Metab. 2, 307–317. 10.1038/s42255-020-0190-0 32601609 PMC7323974

[B4] Brooks M. J. Hajira A. Mohamed J. S. Alway S. E. (2018). Voluntary wheel running increases satellite cell abundance and improves recovery from disuse in gastrocnemius muscles from mice. J. Appl. Physiol. 124, 1616–1628. 10.1152/japplphysiol.00451.2017 29470148 PMC6032091

[B5] Calvani R. Joseph A. M. Adhihetty P. J. Miccheli A. Bossola M. Leeuwenburgh C. (2013). Mitochondrial pathways in sarcopenia of aging and disuse muscle atrophy. Biol. Chem. 394, 393–414. 10.1515/hsz-2012-0247 23154422 PMC3976204

[B6] Cho J. M. Ghosh R. Mookherjee S. Boudina S. Symons J. D. (2022). Reduce, Reuse, Recycle, Run !: 4 Rs to improve cardiac health in advanced age. Aging (Albany NY) 14, 9388–9392. 10.18632/aging.204415 36470665 PMC9792203

[B7] Cho J. M. Park S. K. Ghosh R. Ly K. Ramous C. Thompson L. (2021). Late-in-life treadmill training rejuvenates autophagy, protein aggregate clearance, and function in mouse hearts. Aging Cell 20, e13467. 10.1111/acel.13467 34554626 PMC8520717

[B8] Cho J. M. Park S. K. Kwon O. S. Taylor La Salle D. Cerbie J. Fermoyle C. C. (2023). Activating P2Y1 receptors improves function in arteries with repressed autophagy. Cardiovasc Res. 119, 252–267. 10.1093/cvr/cvac061 35420120 PMC10236004

[B9] Coletta G. Phillips S. M. (2023). An elusive consensus definition of sarcopenia impedes research and clinical treatment: a narrative review. Ageing Res. Rev. 86, 101883. 10.1016/j.arr.2023.101883 36792012

[B10] Da Rocha A. L. Pereira B. C. Pauli J. R. De Souza C. T. Teixeira G. R. Lira F. S. (2016). Downhill running excessive training inhibits hypertrophy in mice skeletal muscles with different fiber type composition. J. Cell Physiol. 231, 1045–1056. 10.1002/jcp.25197 26381504

[B11] Degens H. Turek Z. Hoofd L. Van't Hof M. A. Binkhorst R. A. (1993). Capillarisation and fibre types in hypertrophied m. plantaris in rats of various ages. Respir. Physiol. 94, 217–226. 10.1016/0034-5687(93)90049-g 8272592

[B12] Dungan C. M. Brightwell C. R. Wen Y. Zdunek C. J. Latham C. M. Thomas N. T. (2022). Muscle-specific cellular and molecular adaptations to late-life voluntary concurrent exercise. Funct. (Oxf) 3, zqac027. 10.1093/function/zqac027 PMC9233305 35774589

[B13] Ferrara P. J. Yee E. M. Petrocelli J. J. Fix D. K. Hauser C. T. De Hart N. (2022). Macrophage immunomodulation accelerates skeletal muscle functional recovery in aged mice following disuse atrophy. J. Appl. Physiol. 133, 919–931. 10.1152/japplphysiol.00374.2022 36049060 PMC9550586

[B14] Fix D. K. Ekiz H. A. Petrocelli J. J. Mckenzie A. M. Mahmassani Z. S. O'connell R. M. (2021). Disrupted macrophage metabolic reprogramming in aged soleus muscle during early recovery following disuse atrophy. Aging Cell 20, e13448. 10.1111/acel.13448 34365717 PMC8441489

[B15] Gallegly J. C. Turesky N. A. Strotman B. A. Gurley C. M. Peterson C. A. Dupont-Versteegden E. E. (2004). Satellite cell regulation of muscle mass is altered at old age. J. Appl. Physiol. 97, 1082–1090. 10.1152/japplphysiol.00006.2004 15121742

[B16] Hanson A. M. Stodieck L. S. Cannon C. M. Simske S. J. Ferguson V. L. (2010). Seven days of muscle re-loading and voluntary wheel running following hindlimb suspension in mice restores running performance, muscle morphology and metrics of fatigue but not muscle strength. J. Muscle Res. Cell Motil. 31, 141–153. 10.1007/s10974-010-9218-5 20632203

[B17] Hvid L. Aagaard P. Justesen L. Bayer M. L. Andersen J. L. Ørtenblad N. (2010). Effects of aging on muscle mechanical function and muscle fiber morphology during short-term immobilization and subsequent retraining. J. Appl. Physiology 109, 1628–1634. 10.1152/japplphysiol.00637.2010 20864557

[B18] Joanisse S. Nederveen J. P. Baker J. M. Snijders T. Iacono C. Parise G. (2016). Exercise conditioning in old mice improves skeletal muscle regeneration. FASEB J. 30, 3256–3268. 10.1096/fj.201600143RR 27306336

[B19] Jones R. G. 3rd Dimet-Wiley A. Haghani A. Da Silva F. M. Brightwell C. R. Lim S. (2023). A molecular signature defining exercise adaptation with ageing and *in vivo* partial reprogramming in skeletal muscle. J. Physiol. 601, 763–782. 10.1113/JP283836 36533424 PMC9987218

[B20] Kanazawa Y. Ikegami K. Sujino M. Koinuma S. Nagano M. Oi Y. (2017). Effects of aging on basement membrane of the soleus muscle during recovery following disuse atrophy in rats. Exp. Gerontol. 98, 153–161. 10.1016/j.exger.2017.08.014 28803135

[B21] Kayani A. C. Close G. L. Jackson M. J. Mcardle A. (2008). Prolonged treadmill training increases HSP70 in skeletal muscle but does not affect age-related functional deficits. Am. J. Physiol. Regul. Integr. Comp. Physiol. 294, R568–R576. 10.1152/ajpregu.00575.2007 17989141

[B22] Kim Y. J. Kim H. J. Lee W. J. Seong J. K. (2020). A comparison of the metabolic effects of treadmill and wheel running exercise in mouse model. Lab. Anim. Res. 36, 3. 10.1186/s42826-019-0035-8 32206610 PMC7081706

[B23] Mahmassani Z. S. Mckenzie A. I. Petrocelli J. J. De Hart N. M. Reidy P. T. Fix D. K. (2021). Short-term metformin ingestion by healthy older adults improves myoblast function. Am. J. Physiol. Cell Physiol. 320, C566–C576. 10.1152/ajpcell.00469.2020 33406027 PMC8424538

[B24] Moro T. Brightwell C. R. Phalen D. E. Mckenna C. F. Lane S. J. Porter C. (2019). Low skeletal muscle capillarization limits muscle adaptation to resistance exercise training in older adults. Exp. Gerontol. 127, 110723. 10.1016/j.exger.2019.110723 31518665 PMC6904952

[B25] Olesen A. T. Malchow-Moller L. Bendixen R. D. Kjaer M. Svensson R. B. Andersen J. L. (2021). Age-related myofiber atrophy in old mice is reversed by ten weeks voluntary high-resistance wheel running. Exp. Gerontol. 143, 111150. 10.1016/j.exger.2020.111150 33181317

[B26] Oliveira J. R. S. Mohamed J. S. Myers M. J. Brooks M. J. Alway S. E. (2019). Effects of hindlimb suspension and reloading on gastrocnemius and soleus muscle mass and function in geriatric mice. Exp. Gerontol. 115, 19–31. 10.1016/j.exger.2018.11.011 30448397 PMC6366863

[B27] Padilla C. J. Harrigan M. E. Harris H. Schwab J. M. Rutkove S. B. Rich M. M. (2021). Profiling age-related muscle weakness and wasting: neuromuscular junction transmission as a driver of age-related physical decline. Geroscience 43, 1265–1281. 10.1007/s11357-021-00369-3 33895959 PMC8190265

[B28] Petrocelli J. J. Mahmassani Z. S. Fix D. K. Montgomery J. A. Reidy P. T. Mckenzie A. I. (2021). Metformin and leucine increase satellite cells and collagen remodeling during disuse and recovery in aged muscle. FASEB J. 35, e21862. 10.1096/fj.202100883R 34416035 PMC8384128

[B29] Reidy P. T. Mckenzie A. I. Mahmassani Z. S. Petrocelli J. J. Nelson D. B. Lindsay C. C. (2019). Aging impairs mouse skeletal muscle macrophage polarization and muscle-specific abundance during recovery from disuse. Am. J. Physiol. Endocrinol. Metab. 317, E85–E98. 10.1152/ajpendo.00422.2018 30964703 PMC6689737

[B30] Rhoads R. P. Flann K. L. Cardinal T. R. Rathbone C. R. Liu X. Allen R. E. (2013). Satellite cells isolated from aged or dystrophic muscle exhibit a reduced capacity to promote angiogenesis *in vitro* . Biochem. Biophys. Res. Commun. 440, 399–404. 10.1016/j.bbrc.2013.09.085 24070607

[B31] Schindelin J. Arganda-Carreras I. Frise E. Kaynig V. Longair M. Pietzsch T. (2012). Fiji: an open-source platform for biological-image analysis. Nat. Methods 9, 676–682. 10.1038/nmeth.2019 22743772 PMC3855844

[B32] Scioli M. G. Bielli A. Arcuri G. Ferlosio A. Orlandi A. (2014). Ageing and microvasculature. Vasc. Cell 6, 19. 10.1186/2045-824X-6-19 25243060 PMC4169693

[B33] Seldeen K. L. Lasky G. Leiker M. M. Pang M. Personius K. E. Troen B. R. (2018). High intensity interval training improves physical performance and frailty in aged mice. J. Gerontol. A Biol. Sci. Med. Sci. 73, 429–437. 10.1093/gerona/glx120 28633487

[B34] Smith L. R. Barton E. R. (2014). SMASH - semi-automatic muscle analysis using segmentation of histology: a MATLAB application. Skelet. Muscle 4, 21. 10.1186/2044-5040-4-21 25937889 PMC4417508

[B35] Snijders T. Nederveen J. P. Bell K. E. Lau S. W. Mazara N. Kumbhare D. A. (2019). Prolonged exercise training improves the acute type II muscle fibre satellite cell response in healthy older men. J. Physiol. 597, 105–119. 10.1113/JP276260 30370532 PMC6312443

[B36] Snijders T. Nederveen J. P. Joanisse S. Leenders M. Verdijk L. B. Van Loon L. J. (2017). Muscle fibre capillarization is a critical factor in muscle fibre hypertrophy during resistance exercise training in older men. J. Cachexia Sarcopenia Muscle 8, 267–276. 10.1002/jcsm.12137 27897408 PMC5377411

[B37] Socha M. J. Segal S. S. (2018). Microvascular mechanisms limiting skeletal muscle blood flow with advancing age. J. Appl. Physiol. 125, 1851–1859. 10.1152/japplphysiol.00113.2018 30412030 PMC6737458

[B38] Suetta C. Hvid L. G. Justesen L. Christensen U. Neergaard K. Simonsen L. (2009). Effects of aging on human skeletal muscle after immobilization and retraining. J. Appl. Physiology 107, 1172–1180. 10.1152/japplphysiol.00290.2009 19661454

[B39] Takeshita H. Yamamoto K. Nozato S. Inagaki T. Tsuchimochi H. Shirai M. (2017). Modified forelimb grip strength test detects aging-associated physiological decline in skeletal muscle function in male mice. Sci. Rep. 7, 42323. 10.1038/srep42323 28176863 PMC5296723

[B40] Tanner R. E. Brunker L. B. Agergaard J. Barrows K. M. Briggs R. A. Kwon O. S. (2015). Age-related differences in lean mass, protein synthesis and skeletal muscle markers of proteolysis after bed rest and exercise rehabilitation. J. Physiol. 593, 4259–4273. 10.1113/JP270699 26173027 PMC4594296

[B41] Tidball J. G. Berchenko E. Frenette J. (1999). Macrophage invasion does not contribute to muscle membrane injury during inflammation. J. Leukoc. Biol. 65, 492–498. 10.1002/jlb.65.4.492 10204578

[B42] Tidball J. G. Flores I. Welc S. S. Wehling-Henricks M. Ochi E. (2020). Aging of the immune system and impaired muscle regeneration: a failure of immunomodulation of adult myogenesis. Exp. Gerontol. 145, 111200. 10.1016/j.exger.2020.111200 33359378 PMC7855614

[B43] Triolo M. Oliveira A. N. Kumari R. Hood D. A. (2022). The influence of age, sex, and exercise on autophagy, mitophagy, and lysosome biogenesis in skeletal muscle. Skelet. Muscle 12, 13. 10.1186/s13395-022-00296-7 35690879 PMC9188089

[B44] Walton R. G. Kosmac K. Mula J. Fry C. S. Peck B. D. Groshong J. S. (2019). Human skeletal muscle macrophages increase following cycle training and are associated with adaptations that may facilitate growth. Sci. Rep. 9, 969. 10.1038/s41598-018-37187-1 30700754 PMC6353900

[B45] Wang Y. Wehling-Henricks M. Welc S. S. Fisher A. L. Zuo Q. Tidball J. G. (2019). Aging of the immune system causes reductions in muscle stem cell populations, promotes their shift to a fibrogenic phenotype, and modulates sarcopenia. Faseb J. 33, 1415–1427. 10.1096/fj.201800973R 30130434 PMC6355087

[B46] Welc S. S. Wehling-Henricks M. Antoun J. Ha T. T. Tous I. Tidball J. G. (2020). Differential effects of myeloid cell PPARδ and IL-10 in regulating macrophage recruitment, phenotype, and regeneration following acute muscle injury. J. Immunol. 205, 1664–1677. 10.4049/jimmunol.2000247 32817369 PMC7484367

[B47] White J. R. Confides A. L. Moore-Reed S. Hoch J. M. Dupont-Versteegden E. E. (2015). Regrowth after skeletal muscle atrophy is impaired in aged rats, despite similar responses in signaling pathways. Exp. Gerontol. 64, 17–32. 10.1016/j.exger.2015.02.007 25681639 PMC4359098

[B48] Zhang X. Trevino M. B. Wang M. Gardell S. J. Ayala J. E. Han X. (2018). Impaired mitochondrial energetics characterize poor early recovery of muscle mass following hind limb unloading in old mice. J. Gerontol. A Biol. Sci. Med. Sci. 73, 1313–1322. 10.1093/gerona/gly051 29562317 PMC6132115

