# Supplementary material for: Treadmill training does not enhance skeletal muscle recovery following disuse atrophy in older male mice
Source: Front Physiol. 2023 Oct 24;14:1263500. doi: 10.3389/fphys.2023.1263500 (PMC10628510; doi:10.3389/fphys.2023.1263500)
Supplement: Supplementary file 1 [file Table1.docx]

| Supplemental Table 1. Treadmill training protocol | | | |
| --- | --- | --- | --- |
| Days | Time (min) | Speed (m/min) | Incline (degree) |
| 4 | 20 | 10 | 5 |
| 8 | 30 | 10 | 5 |
| 12 | 45 | 10 | 5 |
| 16 | 60 | 10 | 5 |
| 20 | 60 | 11 | 5 |
| 24 | 60 | 12 | 5 |
| 28 | 30 | 12 | 10 |
| 32 | 45 | 12 | 10 |
| 36 | 60 | 12 | 10 |
| 40 | 60 | 13 | 10 |
| 44 | 60 | 14 | 10 |
| 48 | 45 | 14 | 15 |
| 52 | 60 | 14 | 15 |
| 56 | 60 | 15 | 15 |
| 60 | 60 | 16 | 15 |
| 64 | 45 | 16 | 20 |
| 68 | 60 | 17 | 20 |
| 70 | 60 | 17 | 20 |
