# Supplementary figures and images for: Treadmill training does not enhance skeletal muscle recovery following disuse atrophy in older male mice
Source: Front Physiol. 2023 Oct 24;14:1263500. doi: 10.3389/fphys.2023.1263500 (PMC10628510; doi:10.3389/fphys.2023.1263500)

# Supplemental Figure 2

Tibialis Anterior

A.

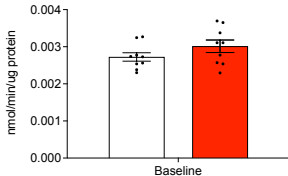

Gastrocnemius

B.

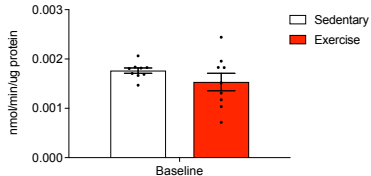

Supplement: Supplementary file 2 [file Image2.pdf]
