# Supplementary material for: Treadmill training does not enhance skeletal muscle recovery following disuse atrophy in older male mice
Source: Front Physiol. 2023 Oct 24;14:1263500. doi: 10.3389/fphys.2023.1263500 (PMC10628510; doi:10.3389/fphys.2023.1263500)
Supplement: Supplementary file 3 [file Table2.docx]

| **Supplemental Table 2.** | **Primer sequences** |  |
| --- | --- | --- |
| **Gene** | **Forward** | **Reverse** |
| CCL2 | 5'-CAGATGCAGTTAACGCCCCA-3' | 5'-TGAGCTTGGTGACAAAAACTACAG-3' |
| IGF-1 | 5'-CACTCATCCACAATGCCTGT- 3' | 5'-TGGATGCTCTTCAGTTCGTG-3' |
| LDHA | 5'-TGTCTCCAGCAAAGACTACTGT-3' | 5'-GACTGTACTTGACAATGTTGGGA-3' |
| PFKP | 5'-GAAACATGAGGCGTTCTGTGT-3' | 5'-CCCGGCACATTGTTGGAGA-3' |
| TGF-β1 | 5'-AACAACGCCATCTATGAGAAAACC-3' | 5'-CCGAATGTCTGACGTATTGAAGAA-3' |
| VEGFc | 5'-GAGGTCAAGGCTTTTGAAGGC-3' | 5'-CTGTCCTGGTATTGAGGGTGG-3' |
