# Supplementary material for: Treadmill training does not enhance skeletal muscle recovery following disuse atrophy in older male mice
Source: Front Physiol. 2023 Oct 24;14:1263500. doi: 10.3389/fphys.2023.1263500 (PMC10628510; doi:10.3389/fphys.2023.1263500)

Supplemental Figure 1

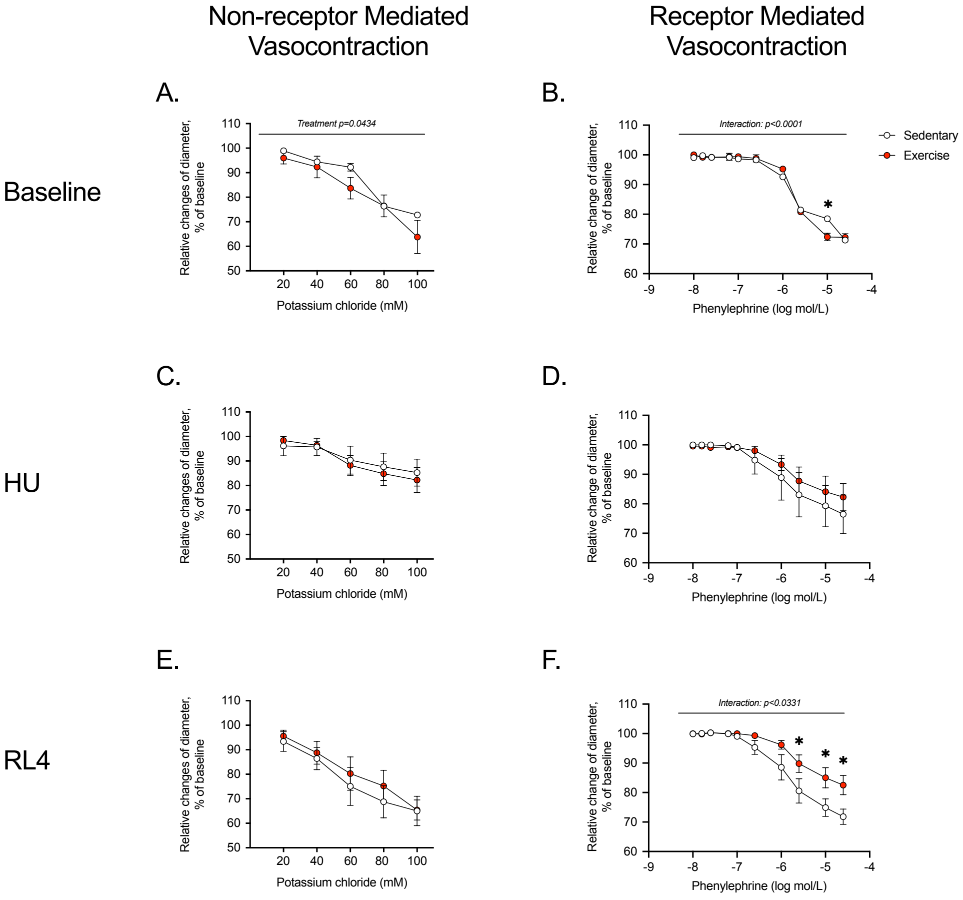

Supplement: Supplementary file 4 [file Image1.pdf]
